# The Involvement of Oxytocin in the Subthalamic Nucleus on Relapse to Methamphetamine-Seeking Behaviour

**DOI:** 10.1371/journal.pone.0136132

**Published:** 2015-08-18

**Authors:** Sarah Jane Baracz, Nicholas Adams Everett, Jennifer Louise Cornish

**Affiliations:** Department of Psychology, Macquarie University, Sydney, Australia, 2109; Washington State University College of Veterinary Medicine, UNITED STATES

## Abstract

The psychostimulant methamphetamine (METH) is an addictive drug of abuse. The neuropeptide oxytocin has been shown to modulate METH-related reward and METH-seeking behaviour. Recent findings implicated the subthalamic nucleus (STh) as a key brain region in oxytocin modulation of METH-induced reward. However, it is unclear if oxytocin acts in this region to attenuate relapse to METH-seeking behaviour, and if this action is through the oxytocin receptor. We aimed to determine whether oxytocin pretreatment administered into the STh would reduce reinstatement to METH use in rats experienced at METH self-administration, and if this could be reversed by the co-administration of the oxytocin receptor antagonist desGly-NH2,d(CH2)5[D-Tyr2,Thr4]OVT. Male Sprague Dawley rats underwent surgery to implant an intravenous jugular vein catheter and bilateral microinjection cannulae into the STh under isoflourane anaesthesia. Rats were then trained to self-administer intravenous METH (0.1 mg/kg/infusion) by lever press during 2-hour sessions under a fixed ratio 1 schedule for 20 days. Following extinction of lever press activity, the effect of microinjecting saline, oxytocin (0.2 pmol, 0.6 pmol, 1.8 pmol, 3.6 pmol) or co-administration of oxytocin (3.6 pmol) and desGly-NH_2_,d(CH_2_)_5_[D-Tyr^2^,Thr^4^]OVT (3 nmol) into the STh (200 nl/side) was examined on METH-primed reinstatement (1 mg/kg; i.p.). We found that local administration of the highest oxytocin dose (3.6 pmol) into the STh decreased METH-induced reinstatement and desGly-NH2,d(CH2)5[D-Tyr2,Thr4]OVT had a non-specific effect on lever press activity. These findings highlight that oxytocin modulation of the STh is an important modulator of relapse to METH abuse.

## Introduction

The psychostimulant methamphetamine (METH) is a potent and addictive illicit drug that is frequently abused worldwide [1]. Chronic METH use can result in serious and pronounced cognitive [2], neurological [3,4] and psychiatric dysfunction [5,6] in addition to physical health problems [7,8]. The reinforcing properties of METH are associated with prolonged and enhanced functionality of the monoamine neurotransmitter dopamine within the mesocorticolimbic circuit [9,10]. Currently, the availability of effective pharmacotherapies for METH dependence is limited [11].

The neuropeptide oxytocin has been proposed as a potential pharmacotherapy for drug dependence due to its strong involvement in modulating both social and drug reward [12,13]. Oxytocin administration has been shown to reduce the rewarding effects and addictive potential of various illicit drugs, one of which being METH [14–20]. In particular, intracerebroventricular (icv) administration of oxytocin prevented the acquisition of a place preference for METH and blunted METH-induced hyperactivity [18]. In addition, Carson et al. [16] showed that intraperitoneal (i.p.) injections of oxytocin reduced the self-administration of METH, reinstatement to METH-seeking behaviour and METH-induced hyperactivity. Further, as social deficits and antisocial behaviour are typically experienced by regular drug users, and oxytocin promotes social engagement and bonding, it has been postulated that in addition to reducing the rewarding effects and abuse potential of various illicit drugs including METH, oxytocin administration could additionally lessen the associated negative social consequences, greatly improving the likelihood of recovery [12,13].

Recently, it was discovered that the subthalamic nucleus (STh) is involved in oxytocin modulation of the cellular and behavioural effects produced by acute METH exposure. Specifically, peripherally administered oxytocin reduced METH-induced cellular activation as measured by Fos expression in the STh [17], and a microinjection of oxytocin into this region attenuated the formation of a conditioned place preference for METH [15].

The STh has only recently been associated with drug and natural reward [17,21–23]. In particular, lesions to the STh decrease motivation for cocaine and alcohol whilst increasing motivation for sucrose rewards [24]. Furthermore, different neuronal populations in this brain region have been shown to selectively respond to cocaine or sucrose reward [22,25], as well as code for reward-related predictions and reward magnitude [21,26]. Considering the published literature, the STh has received little attention for its involvement in reward. In accordance, minimal research has been published examining oxytocin modulation of acute METH reward in the STh [15,17]. Furthermore, no published studies have investigated whether oxytocin is also modulating the effects of chronic exposure to METH within this brain region.

The purpose of the present study was to investigate the ability of oxytocin to modulate reinstatement to METH-seeking behaviour in the STh using the reinstatement model of intravenous METH self-administration. Firstly, we examined whether oxytocin microinjected into the STh would reduce responding on the METH-paired lever when exposed to a METH priming injection after a period of extinction of lever press activity (no METH access). Secondly, we examined if oxytocin modulation of METH lever pressing activity was occurring through the activation of the oxytocin receptor by the concomitant antagonism of oxytocin receptors (OTR) in the STh.

## Materials and Methods

### Animals

32 male Sprague Dawley rats (weighing 200–250 g) were obtained from the Animal Research Centre (Perth, WA, Australia). Rats were housed in pairs (cage size: 40 x 27 x 16 cm until week 6 when they were relocated to larger cages: 64 x 20 x 40 cm) with the exception of a two-day postoperative period of individual housing. Food and water were available *ad libitum* in the home cages and not during experimental procedures. Lighting was kept on a 12-hour light/dark cycle (lights on 06:00), with all experiments conducted during the light cycle. Housing room temperature was maintained at 21°C (±1°C). Prior to the start of experimentation, rats were acclimatised to the facility for seven days and were handled daily for a further seven days. All experimental procedures were conducted in accordance with the Australian Code of Practice for the Care and Use of Animals for Scientific Purposes (7^th^ edition, 2004) and were approved by the Macquarie University Animal Ethics Committee.

### Drugs

Methamphetamine hydrochloride (METH) was purchased from the Australian Government Analytical Laboratories (Pymble, NSW, Australia). Oxytocin was synthesised by AusPep Ltd (Parkville, VIC, Australia). The selective oxytocin receptor (OTR) antagonist desGly-NH_2_,d(CH_2_)_5_[D-Tyr^2^,Thr^4^]OVT was a gift from Dr. Maurice Manning (Department of Biochemistry and Cancer Biology, The University of Toledo, USA). All drugs were dissolved in saline (0.9%) for injection purposes with the oxytocin and OTR antagonist cocktail solutions freshly prepared for each reinstatement session. Vehicle administration was a 0.9% saline solution.

### Apparatus

Testing was conducted in 16 standard operant response chambers (32 x 25 x 34 cm; Med Associates, St Albans, VT, USA), which were housed in sound-attenuating boxes (41 x 56 x 56 cm) equipped with a fan for masking noise and to provide ventilation. Each chamber was equipped with two retractable levers (1 active, 1 inactive) and a house light. The chambers also contained a metal arm with an adjustable weight and a spring connector, which were attached to a swivel. Polyethylene tubing threaded through the spring connector was connected to a 10 ml syringe attached to an infusion pump (Med Associates) located outside of the sound-attenuating chamber. The tubing exiting from the base of the spring connector was connected to the back mount of the intravenous catheter.

Four infrared photobeam detectors were also positioned on the sidewall of each operant chamber to measure locomotor activity. Active and inactive lever presses, number of infusions and locomotor activity was collected and recorded using MED-PC software.

### Surgery

Rats were firstly implanted with a chronic indwelling catheter in the right jugular vein, followed by insertion of bilateral intracranial cannulae to 1mm above the STh. To achieve this, rats were anaesthetised with isoflurane gas (3% in oxygen 2 l/min) and aseptic surgical techniques were used. Catheter implantation, as well as catheter construction is as previously described [27]. Subsequently, rats were placed in a stereotaxic apparatus for bilateral implantation of guide cannulae (26 gauge; 14 mm) to 1 mm above the STh (with nosebar = -3.3 mm, measured from bregma: anterior/posterior, -3.8 mm; lateral, + 2.5 mm; dorsal/ventral, -7.0 mm), similar to our previous study [15]. Co-ordinates were adapted from the rat brain atlas of Paxinos and Watson [28]. Rats were treated with 0.2 ml of the antibiotic cephazolin sodium (100 mg/ml) intravenously and the analgesic carpofen (5 mg/kg) subcutaneously at the time of surgery and daily for the following two days. Following this, catheter patency was maintained by a daily intravenous flush of 0.2 ml of cephazolin sodium in heparinised saline (300 IU/ml). Rats were allowed 5–7 days to recover from surgery before experimentation began.

### Acquisition and maintenance of METH self-administration

Rats were allowed to acquire self-administration of METH during 2-hour fixed ratio 1 scheduled sessions conducted 5 days a week. At the beginning of each session, catheters were flushed with 0.1 ml heparinised saline (10 IU/ml) and were connected to the infusion line. Lever extension and house light illumination indicated the initiation of the session. Levers were allocated as active or inactive, where the location of the active lever was counterbalanced across chambers. Depression of the active lever delivered a 3 s infusion of METH (50 ul, 0.1mg/kg/infusion), immediately followed by the house light extinguishing and a 20 s time out period, during which depression of the active lever was recorded, yet had no consequences. Depression of the inactive lever had no programmed consequences at any time. To avoid overdose, each rat was limited to a maximum of 60 infusions per session. The session ended when either 2 hours had elapsed or the rat had received 60 infusions of METH, and was indicated by lever retraction and the house light turning off. At the end of each session, the infusion line was disconnected and catheters were flushed with 0.2 ml of cephazolin sodium in heparinised saline solution. Self-administration sessions were undertaken for 20 days.

### Extinction

Following the last day of METH self-administration, rats were exposed to daily 2-hour extinction sessions. Depression of the active lever resulted in a saline infusion. Otherwise, the sessions were identical to self-administration sessions. Rats continued under extinction conditions for a minimum of ten days and until < 25 lever presses were made per session for two consecutive days. During the second week of extinction, rats were given one sham saline microinjection into the STh and one i.p. (1 ml/kg saline) injection before the 2-hour session.

### Reinstatement and microinjection procedure

Once extinction criteria were met, rats underwent reinstatement testing. Each reinstatement test session was separated by three extinction days. Prior to reinstatement, rats received a bilateral infusion of treatment or vehicle into the STh (200 nl/side delivered over 1 minute; as previously described in [15]. Both microinjectors (33 gauge; 16 mm) were attached by polyethylene tubing to a 1 μl Hamilton syringe with infusions being driven by a microinjection pump (Harvard Apparatus, USA). The microinjectors remained in position for 30 s after the completion of the microinjection to ensure the entire dose had infused into the brain region. Five minutes following, rats received either a METH (1 mg/kg, i.p.) or vehicle (0.9% saline, i.p.) priming injection and were then placed in the chamber for 2 hours to measure lever pressing activity. Reinstatement conditions were identical to extinction, except for the omission of a max out criteria.

### Experiment treatment conditions

#### Experiment 1

During reinstatement, rats (n = 16) received local administration of one of three doses of oxytocin (0.2 pmol, 0.6 pmol, and 1.8 pmol/side) or vehicle followed by METH (1mg/kg, i.p.). A Latin square design was used to counter ordering effects of treatment. On the last reinstatement test session, rats received a bilateral microinjection of the highest oxytocin dose (1.8 pmol/side) and a vehicle i.p. injection to test if oxytocin alone was unable to reinstate lever press activity. In total, five reinstatement sessions were conducted in each animal.

#### Experiment 2

Experiment 2 was conducted on the basis of local administration of oxytocin into the STh producing a significant reduction in METH-induced reinstatement. Upon identifying a significant reduction, reinstatement testing in experiment 2 involved rats (n = 16) receiving a bilateral microinjection of oxytocin (most effective dose from experiment one), a cocktail of oxytocin and desGly-NH_2_,d(CH_2_)_5_[D-Tyr^2^,Thr^4^]OVT (3 nmol/side) or vehicle followed by a METH i.p. injection. The dose of OTR antagonist used was based on an effective dose to reduce METH reward in the STh from our previous study [14]. On the last reinstatement test session, rats received a bilateral microinjection of the OTR antagonist dose (3 nmol/side) followed by a vehicle i.p. injection to determine that the OTR antagonist alone did not alter reinstatement of lever press activity. In total, four reinstatement sessions were conducted in each animal.

### Histology

Following the completion of testing, rats were deeply anaesthetised with sodium pentobarbitone (135 mg in 1 ml, i.p.) and underwent intracardiac perfusion with 50 ml of 0.9% saline followed by 50 ml of 10% formalin. Brains were extracted, post-fixed in a 10% formalin solution for seven days, and sliced into 60 μm thick coronal sections using a cryostat. Sections were mounted on gel slides. The rat brain atlas of Paxinos and Watson (1997) was used to verify cannulae placement. Only data from rats with correct bilateral cannulae placement were analysed.

### Statistical analysis

Data is displayed as the mean ± SEM. Daily rates of active and inactive lever pressing during self-administration was analysed using a two-way repeated measures analysis of variance (ANOVA). Number of infusions and active lever pressing across the 20-day period were also compared using a repeated-measures ANOVA to ensure rats acquired METH self-administration. Locomotor activity throughout self-administration was analysed using a repeated measures ANOVA. To assess whether rats extinguished METH-paired responses, mean active lever pressing from the last three METH self-administration sessions was compared to active lever pressing during the extinction sessions using a repeated measures ANOVA, as was changes in locomotor activity. Analysis of reinstatement data incorporated the first hour of the two-hour sessions. To determine that rats reinstated METH-paired lever responding, active lever pressing for the first hour of the last day of extinction prior to reinstatement was compared to active lever pressing during the first hour of reinstatement using a two-way repeated measures ANOVA. Active and inactive lever pressing on reinstatement were compared using a two-way repeated measures ANOVA to ensure that the increased lever pressing activity was not due to hyperactivity. Locomotor activity was also analysed across extinction and reinstatement using a two-way repeated measures ANOVA, and across reinstatement sessions using a repeated measures ANOVA. Tests comparing reinstatement test sessions were considered separate a priori hypotheses, and so planned pairwise comparisons were conducted to compare treatment doses to vehicle (experiment 1) as well as to oxytocin treatment (experiment 2; [29,30]. Statistical analyses were performed using SPSS 20 Graduate Student Version for Mac. Statistical significance was set at P < 0.05.

## Results

Of the original 32 rats, 25 completed the study. Two rats did not acquire IVSA, four did not extinguish meth self-administration, and one did not reinstate METH-seeking behavior following METH administration.

### METH self-administration and extinction

**Fig 1:**
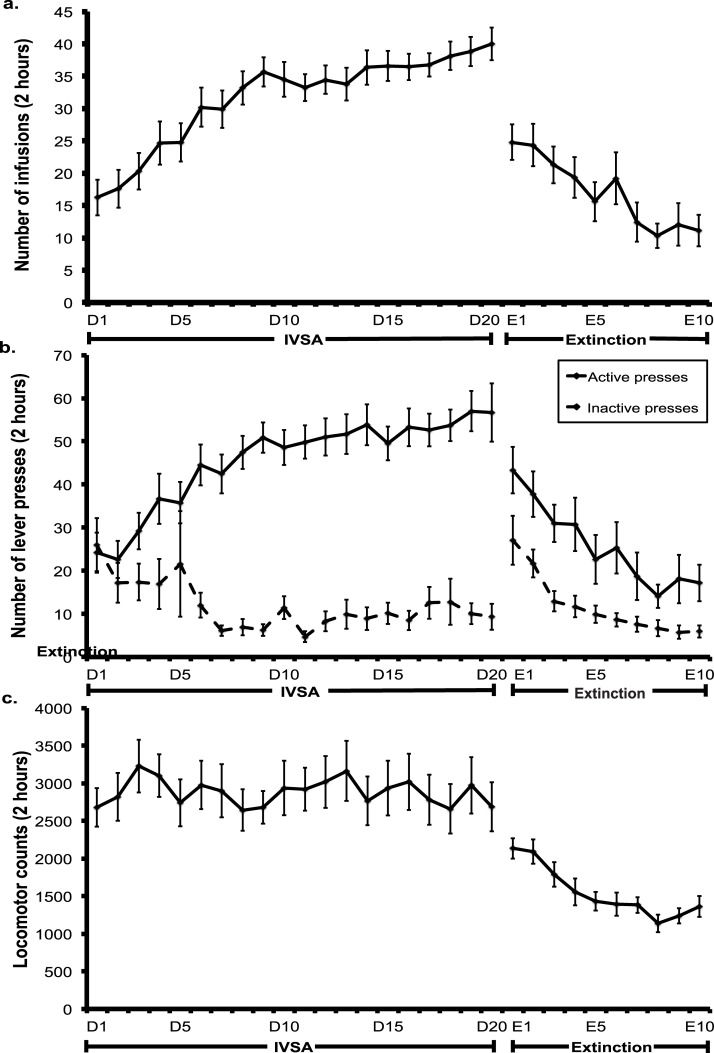
Mean (± SEM) number of a. infusions, b. active and inactive lever presses, as well as c. mean (± SEM) locomotor activity across the 20 days of intravenous METH (0.1mg/kg) self-administration and extinction. Extinction was conducted for a minimum of 10 days and until less than 25 lever presses were made per session for two consecutive days. Only the data from the first 10 days of extinction is displayed in comparison to the mean of the last three days of self-administration.

Analysis of active (*F*(1,18) = 1.526, *p* = 0.233) and inactive lever presses (*F*(1,18) = 0.442, *p* = 0.515) as well as number of infusions (*F*(1,18) = 1.656, *p* = 0.214) during intravenous METH self-administration and active lever presses during extinction (*F*(1,18) = 0.975, *p* = 0.336) showed no significant difference across rat groups in experiment 1 and 2. Additionally, locomotor activity during intravenous METH self-administration (*F*(1,18) = 1.486, *p* = 0.239) and extinction (*F*(1,18) = 0.269, *p* = 0.610) was not significantly different across rat groups experiment 1 and 2. Rats acquired intravenous METH self-administration, as indicated by a significant increase in METH infusions across the 20 day period (day 1 *M* = 15, *SEM* = 3, day 20 *M* = 39, *SEM* = 3; *F*(19,361) = 16.582, *p* < 0.005; see Fig 1a). A significant increase in active lever pressing was also evident (day 1 *M* = 24, *SEM* = 5; day 20 *M* = 55, *SEM* = 7; *F*(19,361) = 8.842, *p* < 0.005). Active and inactive lever pressing was significantly different, indicating rats were able to differentiate between the two levers (*F*(1,19) = 151.937, *p* < 0.005; see Fig 1b). Locomotor activity across the self-administration period was not significantly different across the 20-days (*F*(3.539, 360) = 0.728, *p* = 0.560; see Fig 1c).

Fig 1a and 1b show the mean number of active and inactive lever presses and locomotor activity over the extinction period for rats in both experiment one and two. Throughout the duration of the extinction period, active lever pressing reduced from an average of 43 (*SEM* = 5) on day 1 to 10 (*SEM* = 2) on the last extinction session. Rats in experiment one achieved extinction criteria after 19 sessions, where a significant reduction in active lever pressing, as well as locomotor activity was evident across extinction sessions compared to the average of the last three self-administration sessions (*F*(13, 130) = 17.049, *p* < 0.005; *F*(13, 130) = 5.988, *p* < 0.005 respectively). In experiment two, rats achieved extinction criteria after 13 sessions. A significant reduction in active lever presses, as well as locomotor activity, was present across extinction sessions compared to the mean of the last three self-administration sessions (*F*(16, 368) = 22.021, *p* < 0.005; *F*(16, 128) = 5.823, *p* = < 0.005 respectively).

### Experiment 1

#### Effect of oxytocin on METH-induced reinstatement

**Fig 2:**
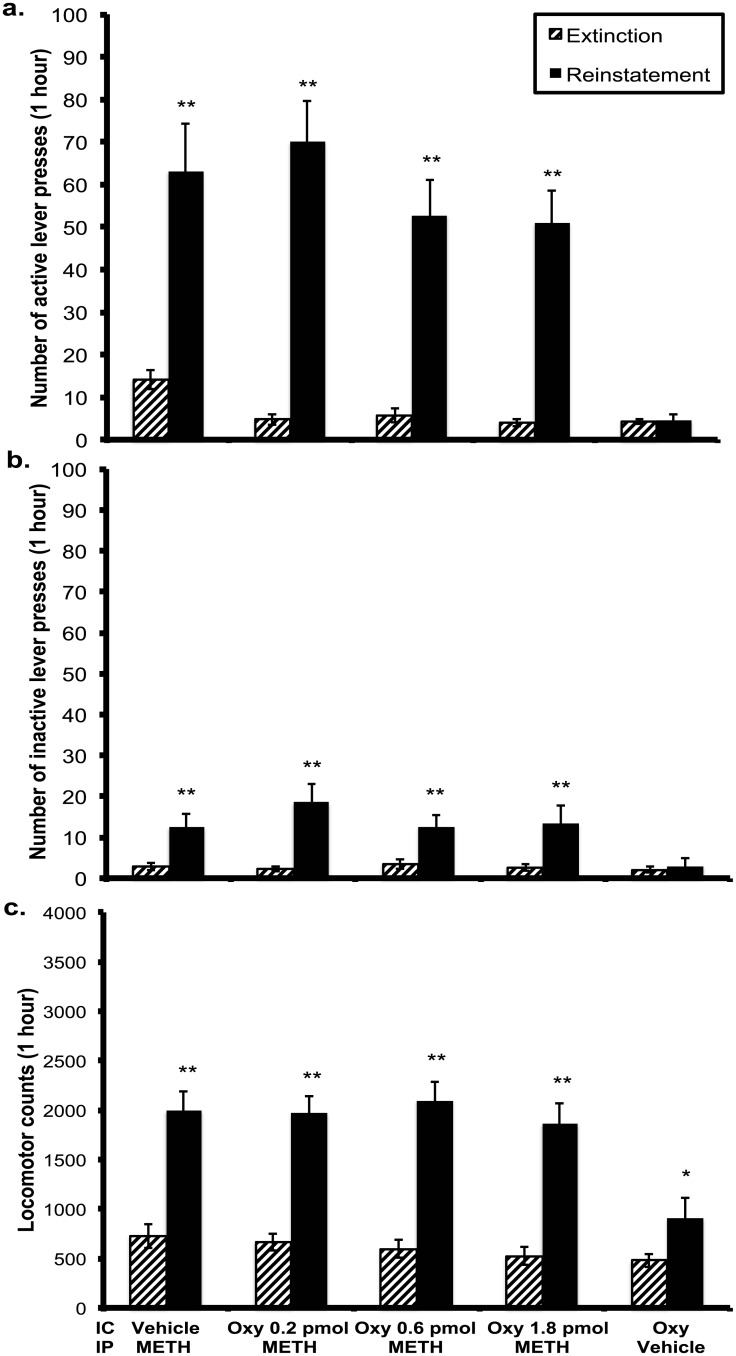
Effects of oxytocin or vehicle microinjection in the STh on a. active lever presses, b. inactive lever presses, and c. locomotor activity during METH (1mg/kg, i.p.) primed reinstatement sessions (n = 8). All animals were exposed to each treatment condition in a counterbalanced manner * p < 0.05, ** p < 0.01 vs prior extinction day. Data are presented as mean ± SEM.

Rats reinstated to their previous active (METH-paired) lever pressing activity following a METH priming injection when compared to active lever pressing on the last extinction session (*F*(1,10) = 29.289, *p* < 0.005; Fig 2a). When considering the microinjection treatments administered prior to the METH priming injection, active lever pressing following the 0.2 pmol, 0.6 pmol and 1.8 pmol/side oxytocin doses were not significantly different to vehicle (*F*(1,10) = 0.522, *p* = 0.486; *F*(1,10) = 2.197, *p* = 0.169; *F*(1,10) = 1.233, *p* = 0.293 respectively). Oxytocin microinjected alone (oxytocin 1.8 pmol/side + vehicle i.p. injection) did not significantly alter active lever pressing compared to the last extinction day (*t*(10) = 0.186, *p* = 0.856). The non-significant effect of oxytocin microinjections to reduce the total number of METH-induced active lever-presses prompted a time-series analysis of the data, with the results included as supplementary information. Briefly, there was an effect of time, although no effect of treatment or a time x treatment interaction occurred.

Inactive lever pressing was significantly higher during reinstatement compared to extinction (*F*(1, 10) = 16.076, *p* = 0.002; Fig 2b), although active lever pressing was significantly higher during reinstatement (*M* = 59.227, *SEM* = 7.242) than inactive lever pressing (*M* = 14.205, *SEM* = 3.211), suggesting rats continued to differentiate between the two levers (*F*(1,10) = 29.289, *p* < 0.005). Inactive lever pressing during reinstatement testing was not significantly different to active lever pressing after oxytocin was solely administered (*t*(10) = 0.887, *p* = 0.396), or to inactive lever pressing on the extinction day prior (*t*(10) = 0.647, *p* = 0.532).

Locomotor activity was significantly higher during reinstatement sessions in comparison to extinction sessions (*F*(1, 10) = 81.755, *p* < 0.005; Fig 2c). Across reinstatement test sessions prior to which a METH prime was administered, locomotor activity was not significantly different (*F*(3, 30) = 1.763, *p* = 0.175). Oxytocin administered alone significantly increased locomotor activity during reinstatement in comparison to the prior extinction session (*t*(10) = 2.577, *p* = 0.028).

### Experiment 2

On the basis of a slight trend in oxytocin reducing METH-induced reinstatement being identified in experiment 1, we tested a higher oxytocin dose of 3.6 pmol/side for the antagonist study. If successful, further reinstatement testing incorporating desGly-NH_2_,d(CH_2_)_5_[D-Tyr^2^,Thr^4^]OVT would be conducted. As such, the first two reinstatement sessions involved counterbalancing of the vehicle and oxytocin microinjections, and on the third session, all rats would receive a cocktail of oxytocin and desGly-NH_2_,d(CH_2_)_5_[D-Tyr^2^,Thr^4^]OVT (3 nmol/side).

#### Effect of higher oxytocin dose and co-administered desGly-NH_2_,d(CH_2_)_5_[D-Tyr^2^,Thr^4^]OVT with oxytocin on METH-induced reinstatement

**Fig 3:**
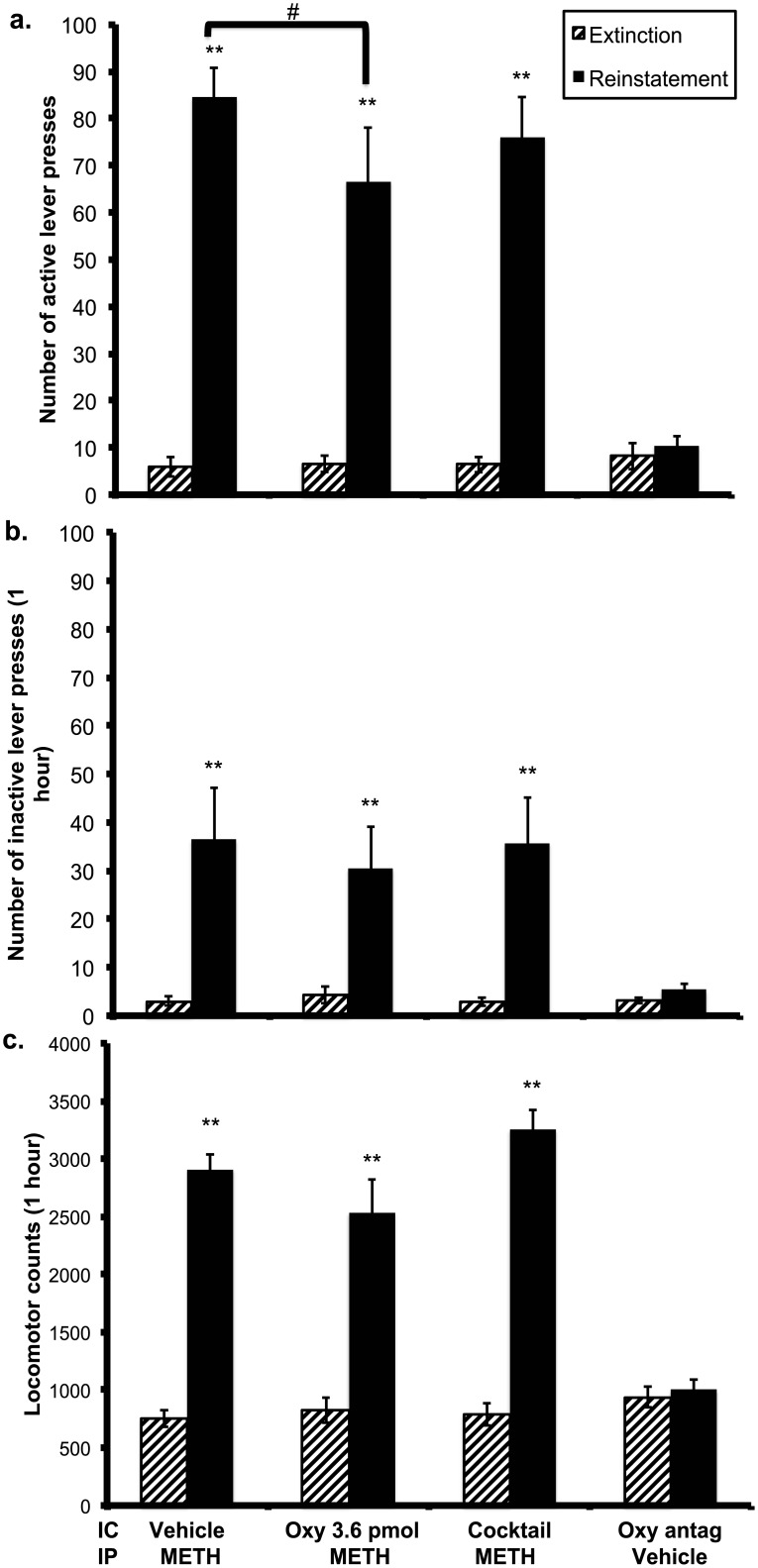
Effects of oxytocin, cocktail, or vehicle microinjection in the STh on a. active lever presses, b. inactive lever presses, and c. locomotor activity during METH (1mg/kg, i.p.) primed reinstatement sessions (n = 8). All animals were exposed to each treatment condition in a counterbalanced manner # p < 0.05 vs. saline + METH condition; ** p < 0.01 vs prior extinction day. Data are presented as mean ± SEM.

Rats lever pressing activity significantly increased during reinstatement sessions following a METH prime compared to the extinction day prior to test (*F*(1, 8) = 83.773, *p* < 0.005; Fig 3a). In comparison to vehicle, a microinjection of the 3.6 pmol/side dose of oxytocin into the STh significantly reduced active lever pressing activity when administered prior to a METH i.p. injection (*F*(1,8) = 6.118, *p* = 0.039). The co-administration of this oxytocin dose with desGly-NH_2_,d(CH_2_)_5_[D-Tyr^2^,Thr^4^]OVT into the STh, however, did not significantly alter METH lever pressing activity when compared to the vehicle microinjection treatment (*F(1*,8) = 1.306, *p* = 0.286). Additionally, the co-administration of desGly-NH_2_,d(CH_2_)_5_[D-Tyr^2^,Thr^4^]OVT with oxytocin was unable to reverse the modulating effect of oxytocin on METH lever pressing activity when compared to the oxytocin microinjection treatment (*F(1*,8) = 1.533, *p* = 0.251). The sole administration of desGly-NH_2_,d(CH_2_)_5_[D-Tyr^2^,Thr^4^]OVT did not significantly alter active lever pressing activity when compared to the extinction day prior (*t*(8) = 0.693, *p* = 0.508). A time-series analysis was also conducted on the experiment 2 data and is included as S1 Text. Briefly, a significant effect of treatment and time was identified, although a time x treatment interaction was not evident.

Inactive lever pressing activity was significantly higher during reinstatement compared to extinction (*F*(1,8) = 16.764, *p* = 0.003; Fig 3b), although active lever pressing was significantly higher (*M* = 75.704, *SEM* = 7.958) than inactive lever pressing (*M* = 34.222, *SEM* = 8.093) on reinstatement suggesting rats continued to differentiate between the two levers (*F*(1,8) = 26.002, *p* = 0.001). Inactive lever pressing was significantly higher when desGly-NH_2_,d(CH_2_)_5_[D-Tyr^2^,Thr^4^]OVT was administered alone on reinstatement in comparison to extinction (*t*(8) = 3.507, *p* = 0.008), although was not significantly different to active lever pressing on reinstatement (*t*(8) = 2.111, *p* = 0.068).

Locomotor activity was significantly higher during reinstatement compared to extinction (*F*(1,8) = 184.158, *p* < 0.005; Fig 3c). Motor activity induced by METH administration was not significantly different across the reinstatement sessions (*F*(1.038,16) = 3.867, *p* = 0.083). DesGly-NH_2_,d(CH_2_)_5_[D-Tyr^2^,Thr^4^]OVT administered alone did not significantly alter locomotor activity throughout reinstatement compared to the prior extinction session (*t*(8) = 0.550, *p* = 0.598).

### Histological analysis

**Fig 4:**
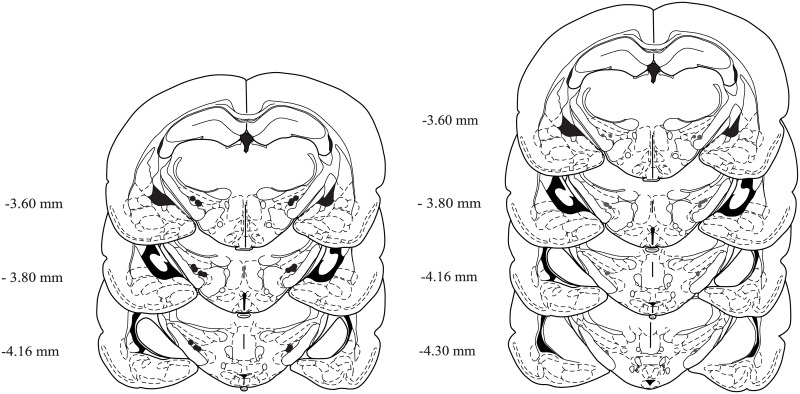
Anatomical coronal diagrams depicting the microinjection sites in the STh. The coronal diagram on the left shows the injection sites for experiment one, and the diagram on the right shows the injection sites for experiment two. The numbers to the left of the image depict the distance in mm from bregma.

Histological examination of cannulae placement mandated the removal of five rats from the sample due to one or both misplaced guide cannulae (three from experiment 1 and two from experiment 2). Fig 4 shows the correctly located cannulae for experiment 1 and 2.

## Discussion

In the present study, oxytocin administered at the highest dose of 3.6 pmol/side into the STh significantly decreased reinstatement to METH-induced lever pressing activity. The co-administration of the selective OTR antagonist with oxytocin, however, did not significantly change METH lever pressing activity when compared to either vehicle or oxytocin treatment. This suggests that oxytocin modulation of relapse to METH-seeking behaviour may be independent of activity at the OTR.

The ability of oxytocin administration to attenuate METH-related behaviours after acute and chronic drug exposure has previously been documented [15,16,18,19]. The STh has also been identified as a key region involved in acute METH and oxytocin interactions [15,17], however the importance of this neuropeptide in regulating the STh in METH-seeking behaviour has not yet been reported. The current study is the first to directly examine the involvement of the STh in mediating the effects of oxytocin on METH-induced reinstatement. At the doses initially examined (0.2 pmol, 0.6 pmol, and 1.8 pmol/side), a significant reduction in lever pressing activity was not identified when oxytocin was microinjected into the STh prior to a METH prime. This result was surprising considering we have previously found the 0.6 pmol/side dose microinjected into the STh to be effective in attenuating the formation of a conditioned place preference to a single pairing with METH [15]. On testing of a higher oxytocin dose (3.6 pmol/side), we found oxytocin to be effective in significantly reducing METH-primed reinstatement.

It has previously been shown that the oxytocin system is dysregulated following chronic illicit drug exposure. You and colleagues [31] identified that rodents chronically exposed to morphine experienced a decrease in oxytocin synthesis in the hypothalamus. Additionally, a decrease of oxytocin content in blood plasma, the hippocampus, and the hypothalamus was evident in rats following a 4-day treatment schedule of twice daily cocaine injections [32]. In the current study we have shown that a dose of oxytocin (0.6 pmol/side) that is effective in preventing acute METH reward [15] did not reduce METH-seeking behaviour in rats that have been exposed to chronic intravenous METH self-administration for 20 days. This suggests that the chronic exposure to METH has likely altered the oxytocin system to necessitate higher doses of oxytocin administration to reduce METH-related behaviours. We are currently investigating the effect of chronic METH self-administration on the oxytocin system and OTR distribution to determine the mechanisms behind this functional change.

To further our understanding of the selectivity of oxytocin activity in the STh on attenuating METH-seeking behaviour, we co-administered the high affinity OTR antagonist desGly-NH_2_,d(CH_2_)_5_[D-Tyr^2^,Thr^4^]OVT with oxytocin [33]. This potent OTR antagonist has been used to determine the involvement of the oxytocin receptor in various behaviours, namely pain modulation [34] and anxiety [35], and has previously been used by our group to determine oxytocin mechanisms in acute METH reward [14] and chronic METH exposure [30].

The co-administration of desGly-NH_2_,d(CH_2_)_5_[D-Tyr^2^,Thr^4^]OVT with oxytocin into the STh produced an interesting outcome. DesGly-NH_2_,d(CH_2_)_5_[D-Tyr^2^,Thr^4^]OVT did not specifically reverse the oxytocin effect on METH-induced lever pressing activity. This was quite surprising considering we have previously examined the effectiveness of this dose in the STh and found it was sufficient to block the modulating effect of oxytocin on the formation of a conditioned place preference for dopamine [14]. However, we have recently reported that the effect of oxytocin administration into the nucleus accumbens to reduce METH-primed reinstatement was also not specifically affected by OTR antagonism [30]. As such, it is likely that OTR functioning has decreased with chronic METH exposure. Indeed, Zanos and colleagues [36] showed that after 10 days of METH i.p. injections in rodents, the OTR was upregulated in the amygdala and hypothalamus, indicating decreased functionality of the receptor. Changes to the functionality or expression of the OTR in the STh offers a potential theory for the limited effect desGly-NH_2_,d(CH_2_)_5_[D-Tyr^2^,Thr^4^]OVT administration had on reversing the oxytocin effect on METH lever pressing activity following chronic METH administration.

The effect of oxytocin to reduce METH-primed reinstatement was not specifically affected by antagonism of the OTR to suggest that oxytocin may be activating alternate receptors beyond the currently identified OTR to modulate METH-induced reinstatement. An additional, currently uncharacterized OTR subtype has been proposed [37,38] and could help explain the inhibitory effect of oxytocin on relapse to METH-seeking behaviour. Alternatively, oxytocin may have acted through vasopressin receptors. Oxytocin is known to bind to vasopressin receptors with reasonable affinity [39] and the vasopressin V1a receptor has been associated with a number of functional effects of oxytocin [40–43]. However, neither the vasopressin V1a or V1b receptors, or the OTR have been localized in the STh using traditional methods of receptor autoradiography, although this is a problematic technique with limited sensitivity [44]. While we have shown a functional effect of oxytocin, through OTR, to reduce METH reward [14] it would clearly be of benefit to examine whether oxytocin is also acting through a functioning V1a receptor in the STh to modulate METH primed reinstatement.

The effects of oxytocin and desGly-NH_2_,d(CH_2_)_5_[D-Tyr^2^,Thr^4^]OVT administration to the STh on METH-seeking behaviour were in the absence of any changes to METH-induced hyperactivity. Methamphetamine administration has consistently been shown to increase locomotor activity in rodents [16,17,19,45]. In line with this, we found that rats exhibited greater locomotor activity during intravenous METH self-administration than in the absence of METH during extinction. Systemic or icv administration of oxytocin has previously been shown to reduce METH-induced hyperactivity [16,17,19]. Our current findings demonstrate that administration of a METH prime increased locomotor activity and that METH hyperactivity was not affected by a microinjection of oxytocin or the co-administration of oxytocin and desGly-NH_2_,d(CH_2_)_5_[D-Tyr^2^,Thr^4^]OVT into the STh. This is in agreement with our previous studies where we examined the involvement of specific brain regions in oxytocin modulation of METH reward and abuse. Namely, we found that oxytocin microinjected into the STh was unable to attenuate METH-induced hyperactivity during the conditioning stage of the conditioned place preference paradigm [15] and that co-administration of desGly-NH_2_,d(CH_2_)_5_[D-Tyr^2^,Thr^4^]OVT with oxytocin into the nucleus accumbens core did not reduce METH-induced hyperactivity on reinstatement to METH-seeking behaviour [30].

Systemically administered oxytocin in the absence of drug administration can cause sedation or no changes to rodent locomotor activity [19,46,47]. Our findings, however showed locomotor activity increased in the oxytocin and vehicle condition when compared to the prior extinction session. This increase in locomotor activity was in the absence of an increase in active or inactive lever pressing. Interestingly, we have previously found that oxytocin solely microinjected into the STh increased locomotor activity in the absence of a conditioned place preference forming [15]. This effect of oxytocin when administered alone is consistent with the lack of effect of oxytocin on METH-induced hyperactivity. Our current findings, in combination with our previous results highlights the likely different neural substrates that are involved in stimulant and reward processes [48].

The STh is in a central position to integrate information from regions that are implicated in chronic drug use, and so greatly influences cognitive and behavioural outcomes. The STh is connected to the mesocorticolimbic circuit through connections with the medial prefrontal cortex and the nucleus accumbens [49], and to the nigrostriatal pathway through connections with the substantia nigra pars compacta and the external segment of the globus pallidus [50]. The STh largely projects to the internal segment of the globus pallidus (GPi) and substantia nigra pars reticulata (SNr); the output nuclei of the basal ganglia [51]. As STh neurons are glutamatergic, excitation of these neurons has an excitatory effect on the GPi and SNr [52]. Neuronal fibres of the output nuclei are GABAergic, therefore their excitation has an inhibitory effect on thalamo-cortical circuits to initiate behaviour [53]. Ultimately, the STh typically suppresses any undesired or inappropriate behaviours through activating the inhibitory fibres of the output nuclei of the basal ganglia. However, we propose that exposure to METH following a period of withdrawal may result in the STh receiving more inhibitory input from the NAc through the activation of D2 receptors as well as inhibitory input from the nigrostriatal pathway [54,55]. Considering that the STh would also be receiving increased dopaminergic input from the ventral tegmental area and the substantia nigra pars compacta [56] and more GABAergic than glutamatergic input, the tonic dopaminergic level would likely be altered, potentially reducing activity in the STh. As the STh would be receiving more inhibitory than excitatory input, this would reduce the control that the STh has over inhibiting the motivation to, and engagement in, seeking and administering METH. It is further proposed then, that local administration of oxytocin into the STh may help recover the normal activity of the region, whereby the STh is able to suppress engagement in compulsive behaviours associated with METH-seeking. However, considering our current findings of the non-specific action of the selective OTR antagonist on blocking the attenuation of METH-primed reinstatement by oxytocin, further investigation into additional receptors activated by oxytocin within the STh, including the V1a receptor [30], will help shed light on the exact role that oxytocin has within the STh as well as direct future examination into the intracellular signaling pathways that are involved in reducing METH-seeking behaviour.

The reduction in METH-related reward and relapse to METH-seeking behaviour following oxytocin administration emphasizes the potential effectiveness of oxytocin as a pharmacotherapeutic treatment for METH dependence. The applicability of oxytocin as a pharmacotherapy also extends to other drugs of abuse as oxytocin administration reduces the abuse and addictive properties of cocaine, opiates, cannabis, and alcohol in rodents [57–60]. Further, oxytocin itself does not elicit rewarding effects following administration [14,15,18], providing additional support for its use as a pharmacotherapy. It is also possible that repeated intranasal oxytocin administration may help restore the dysregulated oxytocin system following drug abuse [61]. Clinical trials examining the effectiveness of intranasal oxytocin administration in human populations abusing various illicit drugs such as cannabis, opiods, and alcohol are currently listed on the National Institute of Health Clinical Trials registry (USA), EU Clinical Trials registry (Europe), and the Australian New Zealand Clinical Trials registry. However, clinical trials examining oxytocin intranasal administration on reducing METH dependence are yet to be listed.

In conclusion, the present study is the first to show that oxytocin microinjected into the STh reduces METH-seeking and that oxytocin modulation of this behaviour does not appear to specifically involve the OTR. The oxytocin effect is specific for METH reward and not METH-induced hyperactivity. These findings highlight an important direction for research into pharmacotherapeutic treatment for METH abuse and addiction.

## Supporting Information

S1 Fig Time-series of non-cumulative active lever pressing for each treatment over 90-minutes of the reinstatement sessions in experiment 1. # *p* < 0.05 Mixed methods ANOVA main effect of time. Error bars have been omitted for clarity.
(DOCX)

S2 Fig Time-series of non-cumulative active lever pressing for each treatment over 90-minutes of the reinstatement sessions in experiment 2. # *p* < 0.05 Mixed methods ANOVA main effect of time, * *p* < 0.05 Mixed methods ANOVA main effect of treatment. Error bars have been omitted for clarity.
(DOCX)

S1 Text (DOCX)

## References

[1] United Nations Office on Drugs and Crime (2010) World Drug Report. Vienna: United Nations

[2] Ornstein TJ, Iddon JL, Baldacchino AM, Sahakian BJ, London M, et al (2000) Profiles of cognitive dysfunction in chronic amphetamine and heroin abusers. Neuropsychopharmacology 23: 114–126. 10.1016/S0893-133X(00)00097-X 10882838

[3] McCann UD, Wong DF, Yokoi F, Villemagne V, Dannals RF, et al (1998) Reduced striatal dopamine transporter density in abstinent methamphetamine and methcathinone users: Evidence from positron emission tomography studies with [C]WIN-35,428. The Journal of Neuroscience 18: 8417–8422. 9763484 10.1523/JNEUROSCI.18-20-08417.1998 PMC6792853

[4] Volkow ND, Chang L, Wang G-J, Fowler JS, Leonido-Yee M, et al (2001) Association of dopamine transporter reduction with psychomotor impairment in methamphetamine abusers. American Journal of Psychiatry 158: 377–382. 11229977 10.1176/appi.ajp.158.3.377

[5] Dyer KR, Cruickshank CC (2007) Depression and other psychological health problems among methamphetamine dependent patients in treatment: Implications for assessment and treatment outcome. Australian Psychologist 40: 96–108.

[6] Harris D, Batki SL (2000) Stimulant psychosis: Symptom profile and acute clinical course. The American Journal of Addictions 9: 28–37. 10.1080/10550490050172209 10914291

[7] Turnipseed SD, Richards JR, Kirk JD, Diercks DB, Amsterdam EA (2003) Frequencey of acute coronary syndrome in patients presenting to the emergency department with chest pain after methamphetamine use. The Journal of Emergency Medicine 24: 369–373. 12745036 10.1016/s0736-4679(03)00031-3

[8] Westover AN, McBride S, Haley RW (2007) Stroke in young adults who abuse amphetamines or cocaine. Archives of General Psychiatry 64. 10.1001/archpsyc.64.4.495 17404126

[9] Koob GF (1992) Drugs of abuse: anatomy, pharmacology and function of reward pathways. Trends in pharmacological sciences 13: 177–184. 1604710 10.1016/0165-6147(92)90060-j

[10] Meredith CW, Jaffe C, Ang-Lee K, Saxon AJ (2005) Implications of chronic methamphetamine use: a literature review. Harvard review of psychiatry 13: 141–154. 16020027 10.1080/10673220591003605

[11] Ciketic S, Hayatbakhsh MR, Doran CM, Najman JM, McKetin R (2012) A review of psychological and pharmacological treatment options for methamphetamine dependence. Journal of Substance Use 17: 363–383.

[12] McGregor IS, Bowen MT (2012) Breaking the loop: Oxytocin as a potential treatment for drug addiction. Hormones and behavior 61: 331–339. 10.1016/j.yhbeh.2011.12.001 22198308

[13] McGregor IS, Callaghan PD, Hunt GE (2008) From ultrasocial to antisocial: a role for oxytocin in the acute reinforcing effects and long-term adverse consequences of drug use? British journal of pharmacology 154: 358–368. 10.1038/bjp.2008.132 18475254 PMC2442436

[14] Baracz SJ, Cornish JL (2013) Oxytocin modulates dopamine-mediated reward in the rat subthalamic nucleus. Hormones and behavior 63: 370–375. 10.1016/j.yhbeh.2012.12.003 23238104

[15] Baracz SJ, Rourke PI, Pardey MC, Hunt GE, McGregor IS, et al (2012) Oxytocin directly administered into the nucleus accumbens core or subthalamic nucleus attentuates methamphetamine-induced conditioned place preference. Behavioural Brain Research 228: 185–193. 10.1016/j.bbr.2011.11.038 22155611

[16] Carson DS, Cornish JL, Guastella AJ, Hunt GE, McGregor IS (2010a) Oxytocin decreases methamphetamine self-administration, methamphetamine hyperactivity, and relapse to methamphetamine-seeking behaviour in rats. Neuropharmacology 58: 38–43. 19560473 10.1016/j.neuropharm.2009.06.018

[17] Carson DS, Hunt GE, Guastella AJ, Barber LL, Cornish JL, et al (2010b) Systemically administered oxytocin decreases methamphetamine activation of the subthalamic nucleus and accumbens core and stimulates oxytocinergic neurons in the hypothalamus. Addiction Biology 15: 448–463. 20731630 10.1111/j.1369-1600.2010.00247.x

[18] Qi J, Yang J-Y, Wang F, Zhao Y-N, Song M, et al (2009) Effects of oxytocin on methamphetamine-induced conditioned place preference and the possible role of glutamatergic neurotransmission in the medial prefrontal cortex of mice in reinstatement. Neuropharmacology 56: 856–865. 10.1016/j.neuropharm.2009.01.010 19371575

[19] Qi J, Yang JY, Song M, Li Y, Wang F, et al (2008) Inhibition by oxytocin of methamphetamine-induced hyperactivity related to dopamine turnover in the mesolimbic region in mice. Naunyn-Schmiedeberg's archives of pharmacology 376: 441–448. 18092152 10.1007/s00210-007-0245-8

[20] Cox BM, Young AB, See RE, Reichel CM (2013) Sex differences in methamphetamine seeking in rats: Impact of oxytocin. Psychoneuroendocrinology 38: 2343–2353. 10.1016/j.psyneuen.2013.05.005 23764194 PMC3775911

[21] Lardeux S, Pernaud R, Paleressompoulle D, Baunez C (2009) Beyond the reward pathway: coding reward magnitude and error in the rat subthalamic nucleus. Journal of neurophysiology 102: 2526–2537. 10.1152/jn.91009.2008 19710371

[22] Baunez C, Dias C, Cador M, Amalric M (2005) The subthalamic nucleus exerts opposite control on cocaine and 'natural' rewards. Nature neuroscience 8: 484–489. 15793577 10.1038/nn1429

[23] Rouand T, Lardeux S, Panayotis N, Paleressompoulle D, Cador M, et al (2010) Reducing the desire for cocaine with subthalamic nucleus deep brain stimulation. Proceedings of the National Academy of Sciences 107: 1196–1200. 10.1073/pnas.0908189107 PMC2824319 20080543

[24] Lardeux S, Baunez C (2008) Alcohol preference influences the subthalamic nucleus control on motivation for alcohol in rats. Neuropsychopharmacology 33: 634–642. 17460610 10.1038/sj.npp.1301432

[25] Lardeux S, Paleressompoulle D, Pernaud R, Cardor M, Baunez C (2013) Different populations of subthalamic neurons encode cocaine versus sucrose reward and predict future error. Journal of neurophysiology 110: 1497–1510. 10.1152/jn.00160.2013 23864369

[26] Darbaky Y, Baunez C, Arecchi P, Legallet E, Apicella P (2005) Reward-related neuronal activity in the subthalamic nucleus of the monkey. NeuroReport 16: 1241–1244. 16012357 10.1097/00001756-200508010-00022

[27] Motbey CP, Clemens KJ, Apetz N, Winstock AR, Ramsey J, et al (2013) High levels of intravenous mephedrone (4-methylmethcathinone) self-administration in rats: Neural consequences and comparison with methamphetamine. Journal of psychopharmacology 27: 823–836. 10.1177/0269881113490325 23739178

[28] Paxinos G, Watson C (1997) The rat brain atlas in stereotaxic co-ordinates. San Diego: Academic Press.

[29] Field A (2009) Discovering statistics using SPSS. London: SAGE Publications.

[30] Baracz SJ, Everett NA, McGregor IS, Cornish JL (in press) Oxytocin in the nucleus accumbens core reduces reinstatement of methamphetamine-seeking behaviour in rats. Addiction Biology. 10.1111/adb.12198 25399704

[31] You ZD, Li JH, Song CY, Wang CH, Lu CL (2000) Chronic morphine treatment inhibits oxytocin synthesis in rats. Neuroreport 11: 3113–3116. 11043533 10.1097/00001756-200009280-00015

[32] Sarnyai Z, Vecsernyes M, Laczi F, Biro E, Szabo G, et al (1992b) Effects of cocaine on the contents of neurohypophyseal hormones in the plasma and in different brain structures iin rats. Neuropeptides 23: 27–31. 1407414 10.1016/0143-4179(92)90006-i

[33] Manning M, Stoev S, Chini B, Durroux T, Mouillac B, et al (2008) Peptide and non-peptide agonists and antagonists for the vasopressin and oxytocin V1a, V1b, V2 and OT receptors: research tools and potential therapeutic agents. Progress in Brain Research 170: 473–512. 10.1016/S0079-6123(08)00437-8 18655903

[34] Yang J, Pan Y-J, Zhao Y, Qiu P-Y, Lu L, et al (2011) Oxytocin in the rat caudate nucleus influences pain modulation. Peptides 32: 2104–2107. 10.1016/j.peptides.2011.08.021 21903147

[35] Figueira RJ, Peabody MF, Lonstein JS (2008) Oxytocin receptor activity in the ventrocaudal periaqueductal gray modulates anxiety-related behavior in postpartum rats. Behavioual Neuroscience 122: 618–628. 10.1037/0735-7044.122.3.618 18513132

[36] Zanos P, Wright SR, Georgiou P, Yoo JH, Ledent C, et al (2014) Chronic methamphetamine treatment induces oxytocin receptor up-regulation in the amygdala and hypothalamus via an adenosine A2a receptor-independent mechanism. Pharmacology, Biochemistry and Behavior 119: 72–79. 10.1016/j.pbb.2013.05.009 23680573

[37] Adan RAH, Van Leeuwen FW, Sonnemans MAF, Brouns M, Hoffman G, et al (1995) Rat oxytocin receptor in brain, pituitary, mammary gland, and uterus: Partial sequence and immunocytochemical localisation. Endocrinology 136: 4022–4028. 7649111 10.1210/endo.136.9.7649111

[38] Chan WY, Wo NC, Stoev S, Cheng LL, Manning M (2003) Discovery and design of novel and selective vasopressin and oxytocin agonists and antagonists: The role of bioassays. Experimental Physiology 85S: 7–18. 10.1111/j.1469-445x.2000.tb00003.x 10795902

[39] Tribollet E, Barberis C, Jard S, Dubois-Bauphin M, Dreifuss JJ (1988) Localization and pharmacological characterization of high affinity binding sites for vasopressin and oxytocin in the rat brain by light microscopic autoradiography. Brain Research 442: 105–118. 2834008 10.1016/0006-8993(88)91437-0

[40] Song Z, McCann KE, McNeill JK IV, Larkin TE II, Huhman L, et al (2014) Oxytocin induces social communication by activating arginine-vasopressin V1a receptors and not oxytocin receptors. Psychoneuroendocrinology 50: 14–19. 25173438 10.1016/j.psyneuen.2014.08.005 PMC4252597

[41] Hicks C, Ramos L, Reekie T, Misagh GH, Narlawar R, et al (2014) Body temperature and cardiac changes induced by peripherally administered oxytocin, vasopressin, and the non-peptide oxytocin receptor agonist WAY 267,464: a biotelemetry study in rats. British journal of pharmacology 171: 2868–2887. 10.1111/bph.12613 24641248 PMC4243861

[42] Ramos L, Hicks C, Kevin R, Caminer A, Narlawar R, et al (2013) Acute prosocial effects of oxytocin and vasopressin when given alone or in combination with 3,4-methylenedioxymethamphetamine in rats: involvement of the V1A receptor. Neuropsychopharmacology 38: 2249–2259. 10.1038/npp.2013.125 23676791 PMC3773675

[43] Caldwell HK, Lee H-J, Macbeth AH, Young WS 3rd (2008) Vasopressin: Behavioural roles of an "original" neuropeptide. Progress in Neurobiology 84: 1–24. 18053631 10.1016/j.pneurobio.2007.10.007 PMC2292122

[44] Freund-Mercier MJ, Stoeckel ME, Dietl MM, Palacios JM, Richard P (1988) Quantitative autoradiographic mapping of neurophypohysial hormone binding sites in the rat forebrain and pituitary gland-I. Characterisation of different types of binding sites and their distribution in the long-evans strain. Neuroscience 26: 261–272. 2843790 10.1016/0306-4522(88)90143-1

[45] Pontieri FE, Crane AM, Seiden LS, Kleven MS, Porrino LJ (1990) Metabolic mapping of the effects of intravenous methamphetamine administration in freely moving rats. Psychopharmacology 102: 175–182. 1980372 10.1007/BF02245919

[46] Shirley DG, Walter MF, Keeler BD, Waters NJ, Walter SJ (2011) Selective blockade of oxytocin and vasopressin V1a receptors in anaesthetised rats: Evidence that activation of oxytocin receptors rather than V1a receptors increases sodium excretion. Nephron Physiology 117: 21–26. 10.1159/000320290 21071981

[47] Uvnas-Moberg K, Ahlenius S, Hillegaart V, Alster P (1994) High doses of oxytocin cause sedation and low doses cause an anxiolytic-like effect in male rats. Pharmacology, Biochemistry and Behavior 49: 101–106. 7816858 10.1016/0091-3057(94)90462-6

[48] Gong W, Justice JJ, Neill D (1997) Dissociation of locomotor and conditioned place preference responses following manipulation of GABA-A and AMPA receptors in ventral pallidum. Progress in Neuro-Pharmacology and Biological Psychiatry 21: 839–852. 10.1016/s0278-5846(97)00084-5 9278955

[49] Chudasama Y, Baunez C, Robbins TW (2003) Functional disconnection of the medial prefrontal cortex and subthalamic nucleus in attentional performance: Evidence for corticosubthalamic interaction. The Journal of Neuroscience 23: 5477–5485. 12843247 10.1523/JNEUROSCI.23-13-05477.2003 PMC6741240

[50] Phillips AG (1984) Brain reward circuitry: A case for separate systems. Brain Research Bulletin 12: 195–201. 6609750 10.1016/0361-9230(84)90189-8

[51] Lintas A, Silkis IG, Alberi L, Villa AE (2012) Dopamine deficiency increases synchronized activity in the rat subthalamic nucleus. Brain Research 1434: 142–151. 10.1016/j.brainres.2011.09.005 21959175

[52] Parent A, Hazrati L-N (1995) Functional anatomy of the basal ganglia. II. The place of subthalamic nucleus and external pallidum in basal ganglia circuitry. Brain Research Reviews 20: 128–154. 7711765 10.1016/0165-0173(94)00008-d

[53] Smith Y, Bevan MD, Shink E, Bolam JP (1998) Microcircuitry of the direct and indirect pathways of the basal ganglia. Neuroscience 86: 353–387. 9881853 10.1016/s0306-4522(98)00004-9

[54] Shen K-Z, Johnson SW (2000) Presynaptic dopamine D2 and muscarine M3 receptors inhibit excitatory and inhibitory transmission to rat subthalamic neurones in vitro. Journal of Physiology 525: 331–341. 10835037 10.1111/j.1469-7793.2000.00331.x PMC2269945

[55] Robinson TE, Berridge KC (1993) The neural basis of drug craving: An incentive-sensitization theory of addiction. Brain research reviews 18: 247–291. 8401595 10.1016/0165-0173(93)90013-p

[56] Shen K-Z, Johnson SW (2000) Presynaptic dopamine D2 and muscarine M3 receptors inhibit excitatory and inhibitory transmission to rat subthalamic neurones in vitro. Journal of Physiology 525: 331–341. 10835037 10.1111/j.1469-7793.2000.00331.x PMC2269945

[57] Kovacs GL, Horvath Z, Sarnyai Z, Faludi M, Telegdy G (1985) Oxytocin and a c-terminal derivative (z-prolyl-d-leucine) attenuate tolerance to and dependence on morphine and interact with dopaminergic neurotransmission in the mouse brain. Neuropharmacology 24: 413–419. 2991800 10.1016/0028-3908(85)90026-7

[58] McGregor IS, Bowen MT (2012) Breaking the loop: Oxytocin as a potential treatment for drug addiction. Hormones and behavior 61: 331–339. 10.1016/j.yhbeh.2011.12.001 22198308

[59] Kovacs GL, Sarnyai Z, Babarczi E, Szabo G, Telegdy G (1990) The role of oxytocin-dopamine interactions in cocaine-induced locomotor hyperactivity. Neuropharmacology 29: 365–368. 2160623 10.1016/0028-3908(90)90095-9

[60] Cui S-S, Bowen RC, Gu G-B, Hanneson DK, Yu PH, et al (2001) Prevention of cannabinoid withdrawal syndrome by lithium: involvement of oxytocinergic neuronal activation. The Journal of Neuroscience 21: 9867–9876. 11739594 10.1523/JNEUROSCI.21-24-09867.2001 PMC6763020

[61] Baskerville TA, Douglas AJ (2010) Dopamine and oxytocin interactions underlying behaviors: potential contributions to behavioral disorders. CNS neuroscience & therapeutics 16: e92–123. 20557568 10.1111/j.1755-5949.2010.00154.x PMC6493805

