# Supplementary material for: The Involvement of Oxytocin in the Subthalamic Nucleus on Relapse to Methamphetamine-Seeking Behaviour
Source: PLoS One. 2015 Aug 18;10(8):e0136132. doi: 10.1371/journal.pone.0136132 (PMC4540453; doi:10.1371/journal.pone.0136132)
Supplement: S1 Text — (DOCX) [file pone.0136132.s003.docx]

**Supplementary information**

**Time-series analysis for experiment 1**

A repeated measures ANOVA was conducted to examine non-cumulative active lever pressing during reinstatement sessions over 30 minute intervals for a total of 90 minutes (S1 Fig.). A significant effect of time was apparent (*F*(2, 20) = 10.758, *p* = 0.004). However, no effect of treatment (*F*(3, 30) = 0.474, *p* = 0.703), or a treatment x time interaction (*F*(6, 60) = 0.222, *p* = 0.893) was evident. In relation to time, active lever pressing was not significantly different in the first 30-minute interval (*M* = 27.36, *SEM* = 3.67) and the second 30-minute interval (30-60 minutes; *M* = 28.71, *SEM* = 3.60; p = 0.487). Although, active lever pressing was higher in the first 30-minutes and the second 30 minute interval (30-60 minutes) compared to the third 30-minute interval (60-90 minutes; *M* = 13.50, *SEM* = 2.31; *p* = 0.010 and *p* = 0.004 respectively). This indicates that the effect of a systemic METH injection is sustained for approximately one hour, and that there was no discernable effect of oxytocin, at any dose tested, on reducing METH lever pressing.

**Time-series analysis for experiment 2**

Fig. S2 shows non-cumulative active lever pressing during reinstatement sessions. A significant treatment (*F*(2, 16) = 5.435, *p* = 0.037) and time (*F*(2,16) = 46.274, *p* < 0.05) effect were evident, although a treatment x time interaction (*F*(4, 32) = 1.147, *p* = 0.352) was not present. Upon further analysis, active lever pressing was higher in the vehicle + METH group (*M* = 30.63, *SEM* = 1.53) compared to the oxytocin + METH group (*M* = 24.00, *SEM* = 3.87; *p* = 0.029). No significant difference in active lever pressing was evident in the vehicle + METH and the cocktail (oxytocin and desGly-NH_2_,d(CH_2_)_5_[D-Tyr^2^,Thr^4^]OVT) + METH groups (*M* = 28.96, *SEM* = 1.78; *p* = 0.127) or the oxytocin + METH and cocktail + METH groups (*p* = 0.075) consistent with the 1-hour data. In terms of time, active lever pressing activity was higher in the first 30-minute interval (*M* = 51.89, *SEM* = 5.01) in comparison to the second 30-minute interval (30-60 minutes; *M* = 23.96, *SEM* = 2.88; *p* <0.05) and the third 30-minute interval (60-90 minutes; *M* = 7.74, *SEM* = 1.84; *p* < 0.05). In addition, active lever pressing activity was higher in the second 30-minute interval (30-60 minutes) than the third 30-minute interval (60-90 minutes; *p* = 0.005). As active lever pressing was still relatively high at the 30-60 minute interval, this does suggest that the effects of a METH i.p. injection are sustained for approximately 1 hour, although in this experiment seem to be more apparent within the first 30 minutes. The data also shows that oxytocin (3.6 pmol/side) microinjected into the STh significantly reduces active lever pressing, although in the absence of an interaction effect, the duration of the oxytocin effect cannot be discerned.
