# Supplementary material for: The Involvement of Oxytocin in the Subthalamic Nucleus on Relapse to Methamphetamine-Seeking Behaviour
Source: PLoS One. 2015 Aug 18;10(8):e0136132. doi: 10.1371/journal.pone.0136132 (PMC4540453; doi:10.1371/journal.pone.0136132)
Supplement: Supplementary file 2 [file pone.0136132.s001.docx]

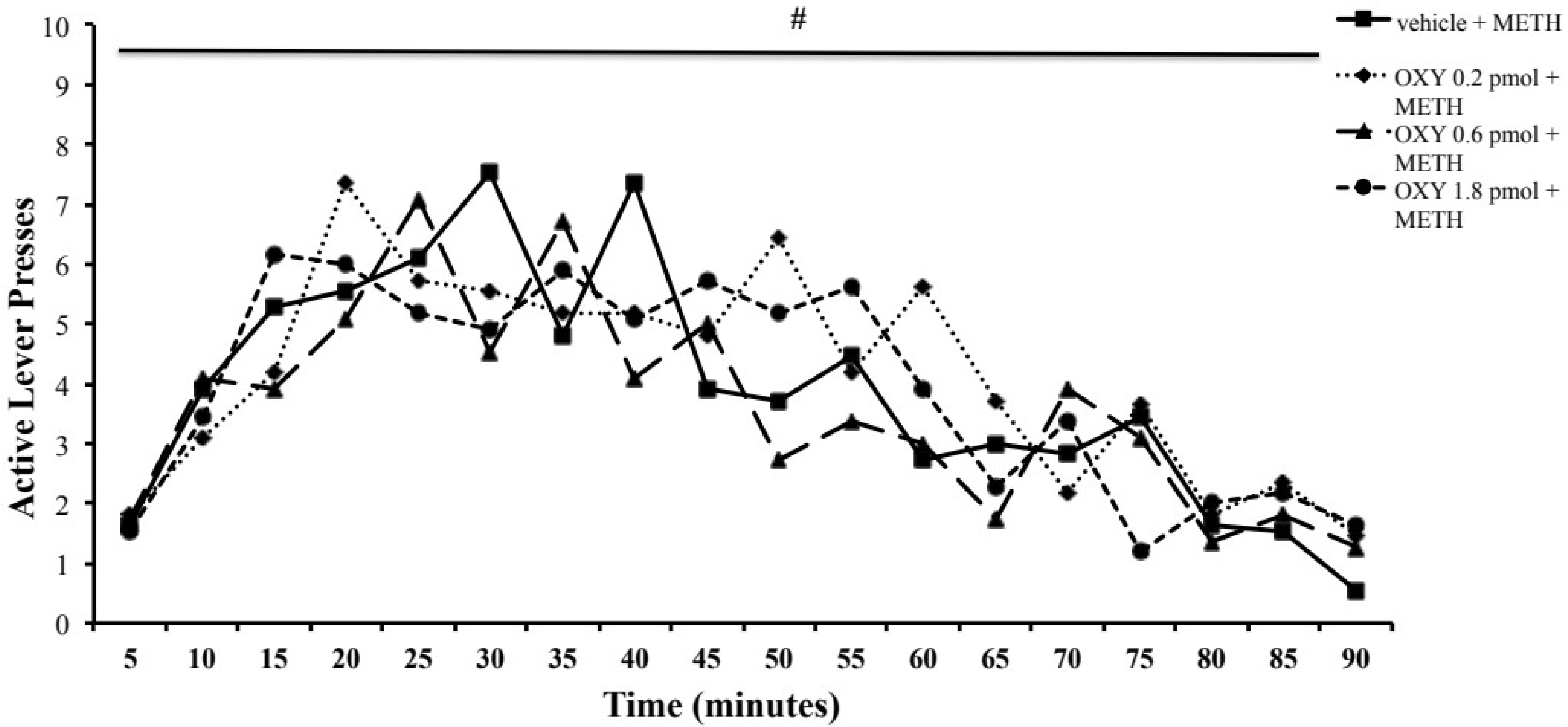
#
Active Lever Presses
0
1
2
3
4
5
6
7
8
9
10
5
10
15
20
25
30
35
40
45
50
55
60
65
70
75
80
85
90
Time (minutes)
vehicle + METH
OXY 0.2 pmol + METH
OXY 0.6 pmol + METH
OXY 1.8 pmol + METH
